# Heterologous Expression and Characterization of Estercin A, a Class II Lanthipeptide Derived from *Clostridium estertheticum* CF016, with Antimicrobial Activity against Clinically Relevant Pathogens

**DOI:** 10.1021/acs.jnatprod.4c00814

**Published:** 2025-01-15

**Authors:** Chenhui Wang, Joseph Wambui, Maria Victoria Fernandez-Cantos, Simon Jurt, Jaap Broos, Roger Stephan, Oscar P. Kuipers

**Affiliations:** † Department of Molecular Genetics, Groningen Biomolecular Sciences and Biotechnology Institute, University of Groningen, Groningen 9747AG, The Netherlands; ‡ Institute for Food Safety and Hygiene, Vetsuisse Faculty, University of Zurich, Zurich CH-8057, Switzerland; § Department of Chemistry, University of Zurich, Zurich CH-8057, Switzerland

## Abstract

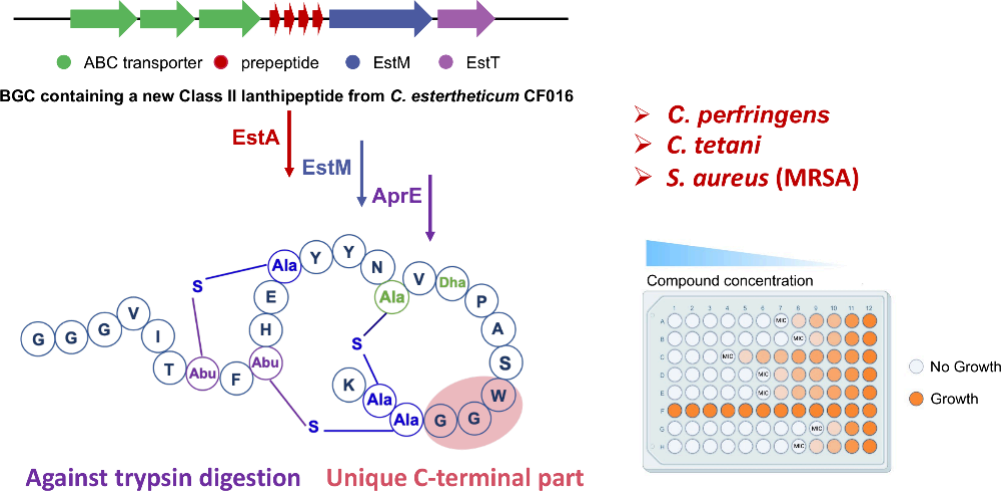

Recent genome mining work revealed that unexplored habitats exhibit great potential for discovering new nonribosomal peptides (NRPs) and ribosomally synthesized and post-translationally modified peptides (RiPPs). Lanthipeptides are a group of RiPPs exhibiting a variety of biological functions. They are characterized by the presence of the thioether-containing bis-amino acids lanthionine and/or methyllanthionine. In this study, we heterologously expressed and structurally characterized estercin A, an unprecedented class II lanthipeptide derived from *Clostridium estertheticum* CF016 in *Escherichia coli*. Comprising 27 amino acids, estercin A features three overlapping (methyl-)lanthionine rings, with a shorter C-terminal part compared to most reported class II lanthipeptides. Estercin A exhibited selective antimicrobial properties against methicillin-resistant *Staphylococcus aureus*, bowel infection-associated *Clostridium perfringens* and *Clostridium tetani*. The mode of action of estercin A was determined as binding to lipid II on the cell membrane. Estercin A exhibited stability across a range of pH values and temperatures and showed resistance to degradation by trypsin. Our findings highlight estercin A as a novel and stable antimicrobial peptide with significant potential in combating clinically relevant pathogens.

Lanthipeptides are ribosomally synthesized and post-translationally modified peptides (RiPPs), which are produced by a wide range of bacteria. They form a significant source of novel bioactive compounds and potential therapeutics. Those having antibacterial activity are best known as lantibiotics.^1−3^ The characteristics of lanthipeptides are the occurrence of lanthionine (Lan) and methyllanthionine (MeLan) residues. These unique ring structures in peptide chains contribute to diverse biological activities and improved stability compared with linear peptides.^4,5^ Lanthipeptides are further subdivided into five classes (I–V) based on differences between the biosynthetic enzymes installing the MeLan ring.^6^ (Me)Lan residues in class II lanthipeptides are installed by the multifunctional enzyme LanM. The N-terminal domain of LanM contains regions that catalyze Ser and Thr dehydration, and the C-terminal region is a LanC-type domain involved in forming the Lan/MeLan ring.^7,8^ The modifications are introduced only in the core peptide, while the N-terminal leader peptide functions as a recognition and binding site for modification enzymes and the ABC transporter (LanT). The leader peptide is proteolytically removed by the peptidase domain (N-terminal 150 residues) of LanT.^6^

Many lanthipeptides have been shown to exhibit antimicrobial activity, antiviral activity, immunomodulatory properties, and antiallodynic effects.^3,9,10^ These properties can be used for several applications. For instance, nisin, the best-studied lanthipeptide, was found to inhibit the growth of several pathogens in food products, such as *Listeria monocytogenes* and *Clostridium tyrobutyricum* and has been widely used as a food preservative.^11,12^ Although some of the currently known lantibiotics have convincing potential to be used as therapeutics, there is a need for improved physiochemical properties, such as higher proteolytic stability, increased specificity against selected pathogens, and an improved production yield. Thus, discovering and evaluating new lantibiotics continues to attract attention. Powerful genome mining methods and tools (e.g., BAGEL4 or antiSMASH) have been developed to guide the discovery and characterization of lanthipeptides.^13−15^

A recent effort to discover novel bacteriocins, including lantibiotics from the psychrophilic *Clostridium estertheticum* complex (CEC), by genome mining has led to the discovery of several bacteriocin biosynthetic gene clusters encoding novel lantibiotics.^16^ Despite this, the identified bacteriocins have not been fully characterized. Estercin A, which was identified in CEC among six putative lanthipeptides, is encoded in the genomes of some strains from three different CEC species, *C. estertheticum*, *C. tagluense* and genomospecies2, whereby each species encodes a different variant of the putative lantibiotic, named as estercin A-T (the position 4 is substituted from Thr to Asn) and estercin A-G (the position 2 is substituted from Gly to Asn). The alignment of the putative estercin A sequence with representative class II lanthipeptides reveals that estercin A is a lacticin 481-like member.^17^ Estercin A shares conserved Cys residues among the lacticin 481 family lanthipeptides on the core peptides, which may indicate a similarity in ring patterns. The core peptide is unique in the C-terminal part, particularly the occurrence of Trp and Gly residues and the replacement of Gly by Asn compared with other peptides in this class are atypical. Trypsin is one of the most common proteolytic enzymes, which specifically cleaves at the C-terminal side of lysine and arginine residues.^18^ The active core of estercin A contains no Arg and one Lys residue at the C-terminus and thus lacks trypsin cleavage sites in the core peptide. These unique features make it an attractive candidate for discovering new lantibiotics with new bioactivities and high resistance against protease digestion.

Usually, natural bacteriocins are extracted from the native producer strains. However, this is constrained by low production yield or even no production.^19,20^ A previous study has shown that it takes up to 6–8 weeks for the slow growing CEC strains to produce cultures ready for RiPPs extraction.^16^ High resolution mass spectrometry (HRMS) and MS/MS analysis for cesin (a characterized lanthipeptide derived from the CEC) also demonstrated that there is a low production of RiPPs in the CEC.^16^ To overcome this challenge, heterologous expression of estercin A genes in an alternative host such as *E*. *coli* is an appealing option. *E. coli* is the most commonly used host for heterologous expression of class II lanthipeptides.^21,22^ Here, we report the successful heterologous expression, production, isolation, characterization, and bioactivity study of the new class II lanthipeptide estercin A that is specifically effective against antibiotic-resistant strains such as *S*. *aureus*, as well as bowel infection-associated *C*. *perfringens* and *C*. *tetani*.

## Results and Discussion

### Heterologous Expression of the Precursor Peptide of Estercin A in *Escherichia coli*

**Figure 1:**
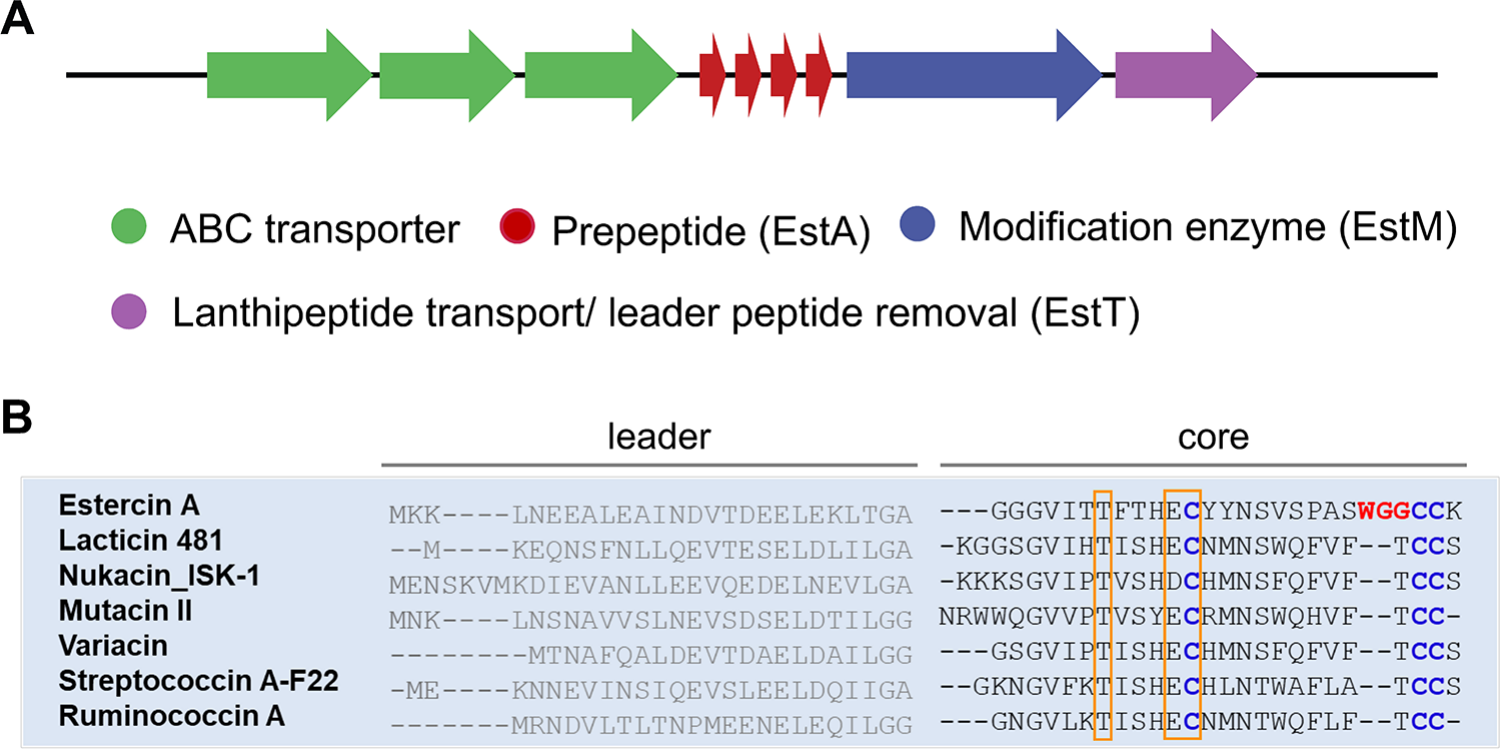
Genetic structure of the estercin A cluster in *C. estertheticum* CF016. Modification enzyme gene (blue) is located downstream of four replicates of the lanthipeptide precursor gene (red). A lanthipeptide transporter gene with a leader removal function (purple) is located downstream of EstM. A group of genes resembling ABC transporters (green) is located at the beginning of the cluster (A). Sequence alignment of estercin A with selected class II lanthipeptides reveals a highly conserved Cys residue, involved in peptide cyclization. The selected class II lanthipeptides lack Trp and Gly in the C-terminal part of the core peptide as well as lacking a Gly preceding the Cys-Cys tandem (B). The lipid II binding motif and identical amino acids in the lantibiotics are highlighted within an orange frame.

Biosynthetic genes involved in the biosynthesis of estercin A were identified in the genome of *C*. *estertheticum* CF016. The three genes, termed *estA*, *estM*, *estT*, were identified as homologues to essential biosynthetic genes found in class II lanthipeptide biosynthesis (Figure 1). The gene *estA* (4 gene copies) encodes the precursor peptide, which is composed of a 26-amino-acid-residue leader peptide with a predicted Gly-Ala cleavage site, and a 27-amino-acid core peptide that includes three Thr residues, three Ser residues and three Cys residues. The presence of multiple copies of structural genes is also observed for other lanthipeptides, which may result in increased expression levels of lanthipeptides in the native strains or have potential evolutionary significance.^23−25^ The *estM* gene encodes a putative LanM-type modification enzyme that includes an N-terminal dehydratase domain and a C-terminal cyclase domain, suggesting that EstM is involved in the ring formation by performing both dehydration and cyclization. EstT is predicted to be an ABC transporter with an N-terminal peptidase domain and a C-terminal ATP-binding domain, indicating that EstT is capable of cleaving off the leader peptide and transport of the mature peptide across the cell membrane. BLAST analysis suggests that the three ABC transporters highlighted in green are likely to comprise ABC transporter permeases and ATP-binding proteins, which may function cooperatively to facilitate the translocation of lanthipeptides across the cell membrane.

The isolation of active RiPPs directly from a CEC strains fermentation experiment under standard lab conditions has been shown to be inefficient and time-consuming.^16^ Previous studies have demonstrated the feasibility of the heterologous expression of entire biosynthetic gene clusters (BGCs) in Gram-positive hosts, including the production of nisin and nukacin ISK-1 in *Lactococcus lactis* and subtilin in *Bacillus subtilis*.^26−28^ Nevertheless, cloning full BGCs often encounters regulatory incompatibilities in the new host.^29^ For well-characterized clusters, such as those associated with nisin and subtilin, heterologous expression of the full gene set in host-related strains typically entails minimal risk. However, for newly identified lanthipeptide BGCs, particularly those originating from underexplored strains, adopting a minimal gene set approach that includes only essential biosynthetic genes can enhance production efficiency, resulting in a more streamlined biosynthetic process. This approach has been successfully applied in a number of previously reported RiPPs genome-mining studies.^21,22,30,31^

**Figure 2:**
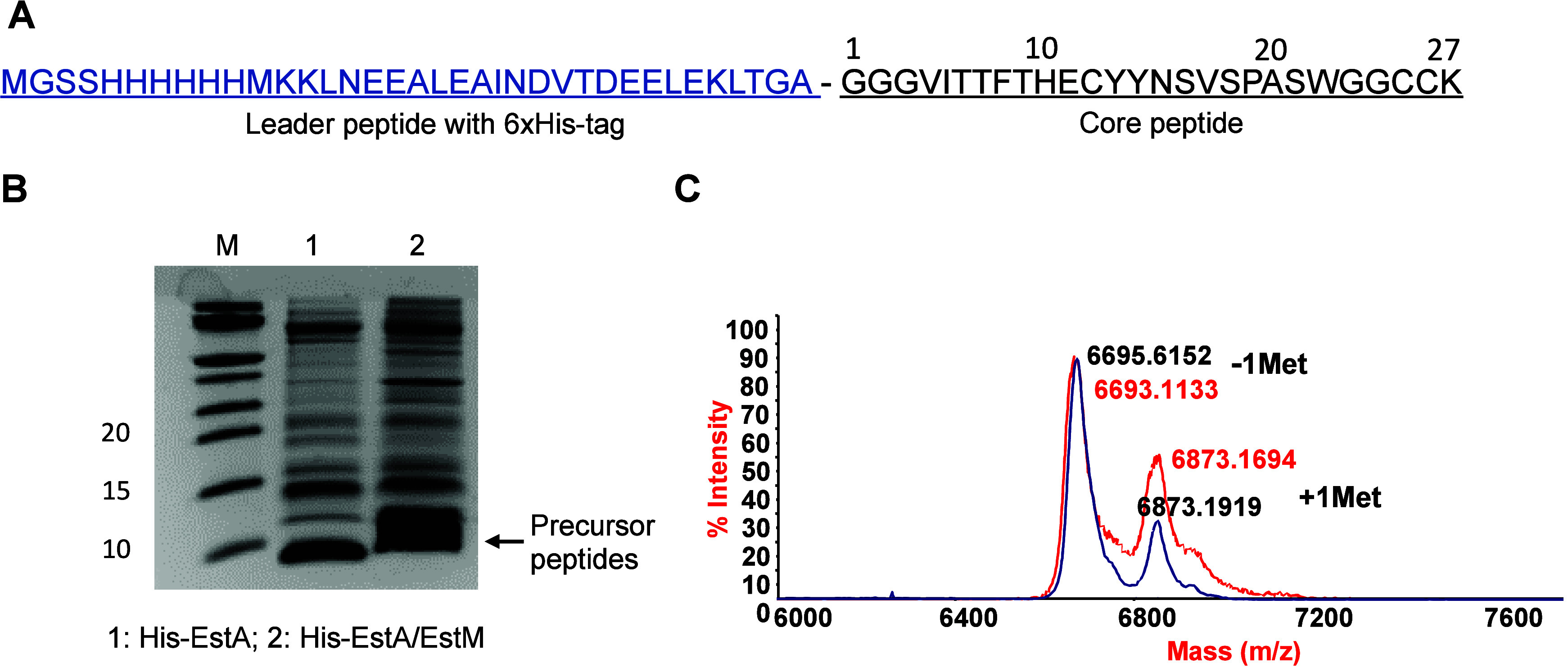
Sequence design for heterologous expression of estercin A prepeptide in *E. coli* BL21(DE3) (A). Tricine-SDS-PAGE of the linear prepeptide and modified prepeptide (B). MALDI-TOF MS analysis of the prepeptide. The most prominent peak mass (blue) was 6695.6 Da compared to a predicted mass of 6698.2 Da of a modified prepeptide with four dehydrations. *N*-Ethylmaleimide (NEM) alkylation assay to determine the pattern of cyclization. The mass of the major peaks after addition of NEM (red) was consistent with the mass of the prepeptide before the reaction with NEM (blue) confirming all Cys residues are involved in ring formation (C). The most prominent molecular weight in the spectrum of each peptide corresponds to the loss of the N-terminal Met, while the second most prominent molecular weight corresponds to the retention of the N-terminal Met.

This study employed the heterologous expression of essential biosynthetic genes in *E*. *coli* to produce the pre-estercin A, combined with subsequent *in vitro* proteolytic leader removal. A modular expression system was constructed to achieve the expression of pre-estercin A in *E. coli* BL21(DE3). The N-terminally His-tagged estercin A precursor His-EstA (Figure 2A) was coexpressed with or without *estM* to explore the dehydrations and the ring formation. After lysis of the cell pellet, precursor peptides were purified using His-tag purification chromatography and desalted by the C_18_ open column. The tricine-sodium dodecyl sulfate-polyacrylamide gel electrophoresis (tricine-SDS-PAGE) result showed that both His-EstA and His-EstA/EstM were successfully produced (Figure 2B). Subsequently, the matrix-assisted laser desorption ionization–time-of-flight mass spectrometry (MALDI-TOF MS) was performed to detect the molecular weights. The mass results showed that compared with His-EstA, His-EstA/EstM exhibited a most prominent mass (−72.04 Da) corresponding to four dehydrations (Figure 2C). Next, an *N*-ethylmaleimide (NEM) assay was performed to detect existing free cysteines in the peptide. Compared with the main mass (6695.62 Da) before the NEM reaction, the main mass (6693.11 Da) after the NEM reaction demonstrated that His-EstA/EstM did not react with NEM (Figure 2C), indicating that all three Cys residues located in the core peptide participated in the formation of the three (Me)Lan rings. Applying the heterologous expression system described here, modified pre-estercin A was purified from the Gram-negative host *E. coli* BL21(DE3). Considering the time-consuming and multifactor induced efforts of improving the culturing conditions using the *C. estertheticum* CF016 as an estercin A-producing strain, the strategy of heterologous expression in *E. coli* provides an effective way to produce the new lanthipeptide. Also, this makes it possible to generate bioengineering variants, because there are currently no available genetic engineering tools for CEC strains.

### Structural Characterization of the Lanthipeptide Estercin A

In order to remove the leader part and obtain the active core peptide for further tandem mass spectrometric analysis (ESI-Q-TOF MS-MS) and an antimicrobial activity test, we first tried heterologously expressing a construct including the first 150 amino acids of the N-terminal region of EstT labeled with a His-tag in *E. coli* which is supposed to be the protease domain responsible for the leader removal. Expression of this His-tagged protease was confirmed by SDS-PAGE (data not shown). Next, the produced peptide His-EstA/EstM was incubated with the purified protease at 37 °C for 3 h. However, the mass result showed that the leader was not removed (Supporting Information (SI), Figure S1B). This suggests the peptidase domain of EstT was not functionally expressed in *E. coli*. The leader peptides of class II lantibiotics typically contain an ELXXBX motif (where B represents Val, Leu or Ile) and generally terminate in a conserved double Gly motif (GG/GA/GS).^32^ The sequence of estercin A follows this pattern (Figure 1B), with a GA motif located after the ELEKLT sequence. Based on this structure, the GA motif can be considered a reliable prediction for the cleavage site of the natural precursor peptide. Next a protease is needed to cleave at this cleavage site and we explored the use of the extracellular *Bacillus amyloliquefaciens* protease AprE,^33,34^ which is known to activate lanthipeptides like presubtilin, premersacidin and prebalucin.^31,34,35^ This protease can cleave a peptide at an Ala residue (C-terminal side) (other cleavable residues include Met, Lys, Asp, Arg, Gln and Phe).^36^ We mixed purified AprE with pre-estercin A *in vitro*, and mass spectrometry analysis indeed showed that the leader removal was achieved exactly behind the predicted GA site (SI, Figure S1C). In summary, an experimental approach is presented to cleave pre-esterin A successfully at the predicted cleavage site with protease AprE. It is highly likely that the active core peptide produced is identical to the natural form of estercin A.

**Figure 3:**
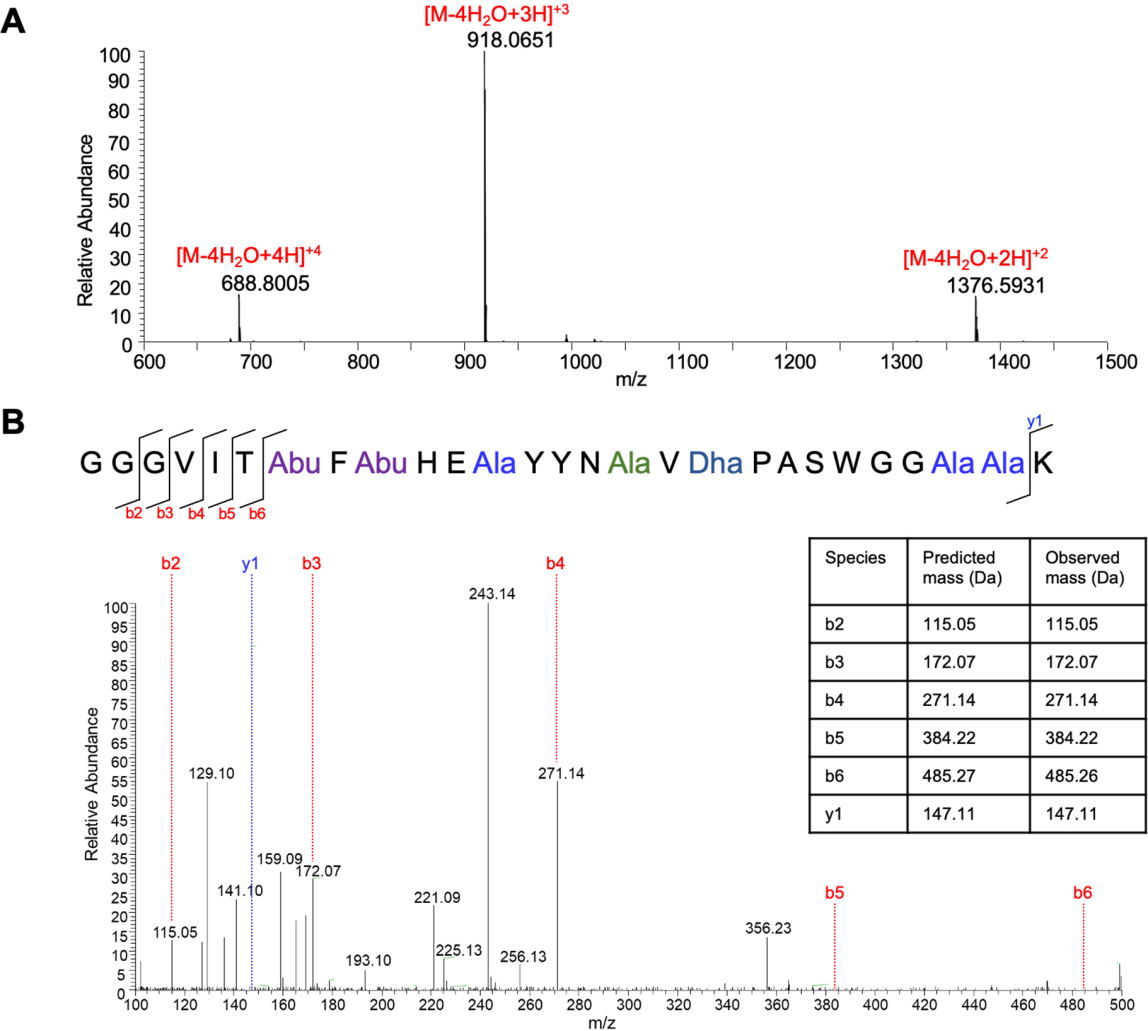
Masses of peptide ions found in LC-MS for estercin A core peptide and the corresponding dehydration numbers (monoisotopic mass, [M-4H_2_O+4H]^+4^, calcd *m*/*z* 688.7990, obsd *m*/*z* 688.8005; [M-4H_2_O+3H]^+3^, calcd *m*/*z* 918.0629, obsd *m*/*z*; 918.0651; [M-4H_2_O+2H]^+2^, calcd *m*/*z* 1376.5907, obsd *m*/*z* 1376.5931) (A). MS/MS fragmentation of estercin A (B).

Subsequently, the core peptide of estercin A was subjected to liquid chromatography–mass spectrometry (LC-MS) analysis, which showed [M-4H_2_O+4H]^+4^ ion at *m*/*z* 688.8005 (calculated *m*/*z* value, 688.7990), [M-4H_2_O+3H]^+3^ ion at *m*/*z* 918.0651 (calculated *m*/*z* value, 918.0629), and [M-4H_2_O+2H]^+2^ ion at *m*/*z* 1376.5931 (calculated *m*/*z* value, 1376.5907) (Figure 3A). Next the protonated peptide was fragmented by collision-activated dissociation and the tandem mass spectrometry (MS/MS) spectrum is shown in Figure 3B. Peptide fragmentation yields b and y ions but the (Me)Lan modification prevents fragmentation within the ring. No credible masses corresponding to fragment ions generated by cleavage between Thr7 and Cys26 where observed, which suggested the existence of cross-ring structures between them. Comparison of the sequence of estercin A with the well-studied lacticin 481 showed that they share conserved Cys residues and conserved positions for dehydrated residues, indicating a similar ring formation in estercin A as in lacticin 481(Figure 1B).

**Figure 4:**
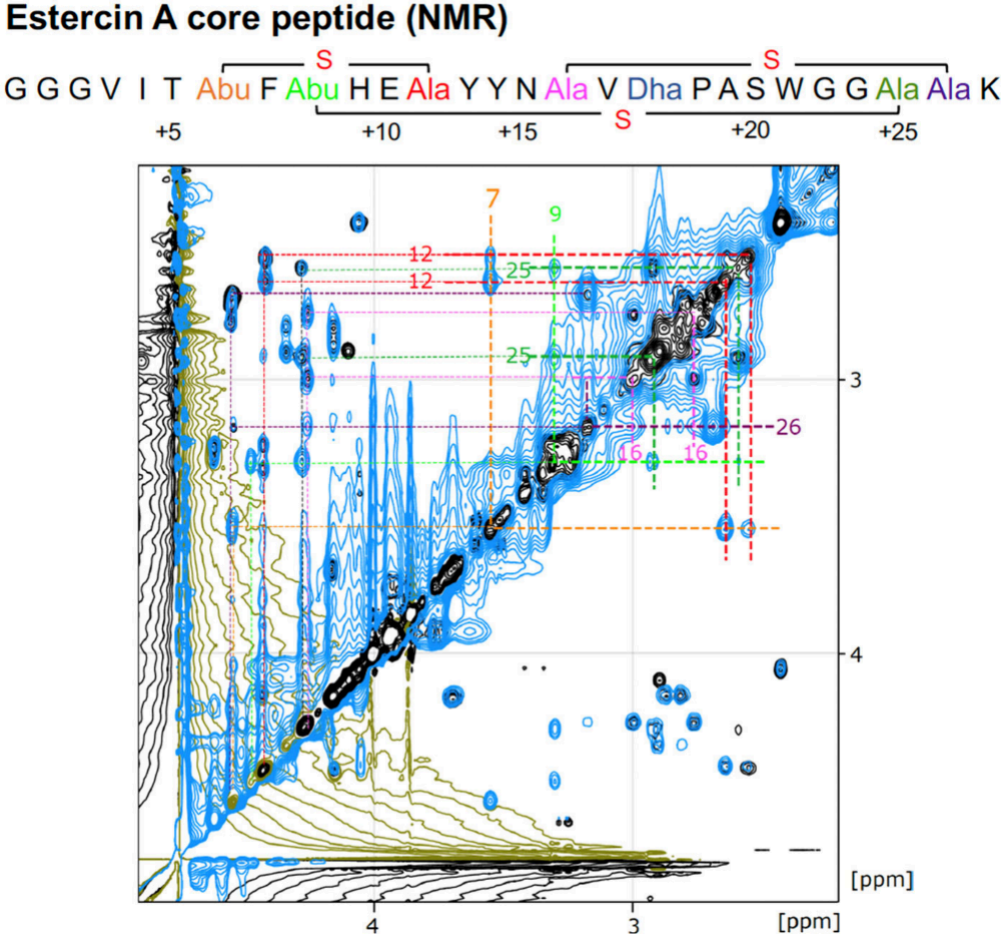
NMR analysis of the core peptide of estercin A. Overlay of two-dimensional NMR spectra (TOCSY in black, NOESY in blue) in the aliphatic region, emphasizing CH_2_–S–CH_2_ intrabridge correlations for deducing the Lan/MeLan ring pattern. Residues are colored based on the sequence diagram above. The NMR data also reveals the dehydration of Ser18 to Dha. Refer to Table 1 and the Supporting Information (SI, Figures S2 and S3) for complete structural assignment data.

To confirm the internal structure of the core peptide, the sample was further subjected to NMR analysis. Cysteine cross-linking was established based on interresidue Hβ-Hβ NOEs and supported by the significant upfield shifts observed for the carbon and proton nuclei in the β-position of Thr 7, Thr 9, and Ser 16 (Figure 4). Through this approach, three (methyl-)lanthionine linkages were identified: the first between Dhb7 (formerly Thr7) and Cys12, the second between Dha9 (formerly Ser9) and Cys25, with this ring crossing the first ring, and the third between Dha16 (formerly Ser16) and Cys26. NMR data also clearly demonstrated that one Dha, which was not involved in the ring formation, is at position 18. The high chemical shift of CA (138.1 ppm) indicates the presence of conjugated double bonds. The high chemical shift of H (10.06 ppm) is also characteristic of Dha due to these double bonds. The elevated chemical shifts of Hβ (4.89 and 5.23 ppm) further support the conclusion that the 18th residue is Dha based on these NMR data characteristics (Table 1). Thus, the structure of synthetic estercin A could be unambiguously established using MS and NMR techniques (Figure 4).

**Table 1:** Chemical Shift Assignments for Estercin A Core Peptide

residue	CA (ppm)	H (ppm)	Hα (ppm)	Hβ (ppm)	others
Gly (1)	43.4		3.85		
Gly (2)	45.1	8.58	4.00		
Gly (3)	45.0	8.37	3.93		
Val (4)	62.3	8.04	4.10	2.00	Cβ: 32.9, Cγ1: 20.6, Cγ2: 21.1, Hγ1: 0.89, Hγ2: 0.87
Ile (5)	60.8	8.42	4.27	1.84	Cβ: 38.6, Cγ1: 27.1, Cγ2:17.9, Cδ1: 12.7, Hδ1: 0.78, Hγ1x: 1.46, Hγ1y: 1.13, Hγ2: 0.86
Thr (6)	62.0	8.37	4.43	4.15	Cβ: 70.3, Cγ2: 21.5, Hγ2: 1.19
Thr (Lan; 7)	60.9	7.91	4.55	3.55	Cβ: 46.6, Cγ2: 22.4, Hγ2: 1.22
Phe (8)	57.5	8.00	4.90	2.93	Cβ: 41.1, Cδ: 132.0, Cε: 131.5, Cζ: 129.9, Hδ: 7.10, Hε: 7.21, Hζ: 7.16
Thr (Lan; 9)	59.7	7.82	4.48	3.30	Cβ: 43.0, Cγ2: 21.9, Hγ2: 0.86
His (10)	58.0	9.11	4.43	3.32/3.24	Cβ: 28.4, Cδ2: 120.4, Cε1: 136.5, Hδ2: 7.30, Hε1: 8.53
Glu (11)	58.0	8.61	4.06	2.11	Cβ: 28.2, Cγ: 34.3, Hγ: 2.43
Cys (Lan; 12)	55.9	7.09	4.42	2.64/2.55	Cβ: 38.6
Tyr (13)	58.3	7.62	4.34	2.89/2.81	Cβ: 38.1, Cδ: 133.4, Cε: 118.2, Hδ: 7.05, Hε: 6.81
Tyr (14)	58.2	7.58	4.16	2.87/2.81	Cβ: 37.9, Cδ: 133.2, Cε: 118.3, Hδ: 6.89, Hε: 6.76
Asn (15)	53.5	7.82	4.55	2.79/2.73	Cβ: 38.3, Hδ2x: 7.49, Hδ2y: 6.84
Ser (Lan; 16)	57.5	7.88	4.25	2.99/2.77	Cβ: 35.5
Val (17)	62.0	7.76	4.12	2.13	Cβ: 32.9, Cγx: 21.3, Cγy: 20.4, Hγx: 0.91, Hγy: 0.87
Ser (Dha; 18)	138.1	10.06		4.89/5.23	Cβ: 109.0
Pro (19)	63.7		4.05	2.15/1.79	Cβ: 32.2, Cγ: 27.0, Cδ: 52.5, Hδx: 3.41, Hδy: 3.34, Hγ: 1.73
Ala (20)	53.0	7.89	4.16	1.23	Cβ: 19.1
Ser (21)	59.7	7.74	4.17	3.69	Cβ: 63.2
Trp (22)	57.7	7.69	4.62	3.28/3.24	Cβ: 29.0, Cδ1: 127.1, Cε3: 120.9, CH2: 124.71, Cζ2: 114.57, Cζ2: 122.0, Hδ1: 7.19, Hε1: 10.10, Hε3: 7.55, Hη2: 7.17, Hζ2: 7.42, Hζ3: 7.09
Gly (23)	45.7	7.95	3.93/3.75		
Gly (24)	46.0	7.92	3.93		
Cys (Lan; 25)	55.7	7.94	4.28	2.92/2.59	Cβ: 35.2
Cys (Lan; 26)	55.0	8.11	4.54	3.17/2.68	Cβ: 36.4
Lys (27)	57.3	7.75	4.10	1.63/1.73	Cβ: 33.6, Cγ: 24.7, Cδ: 29.1, Cε: 42.1, Hδ: 1.58, Hε: 2.89, Hγ: 1.29, Hζ: 7.44

To further validate an additional dehydration outside of the Lan/MeLan rings originates from Ser18, we constructed two mutants: S18A and S21A. MALDI-TOF MS analysis showed that S21A (6677.44 Da) formed 4 dehydrations as the prepeptide of estercin A while S18A (6696.19 Da) formed 1 dehydration less (SI, Figure S4), meaning that the Ser residue at position 18 was modified by EstM, while the Ser at position 21 was not. Additionally, compared with most class II lanthipeptides, we found two unique parts in estercin A: the lack of Trp and Gly as well as Gly instead of Thr flanking the N-terminal site of the double Cys residues (Figure 1B). The relationship between the C-terminal part of estercin A and its antimicrobial activity will be presented below.

### Estercin A Displays Potent Antimicrobial Activity Against Selected Pathogenic Strains

Estercin A was structurally identified as a lacticin 481-like lanthipeptide and would, therefore, be expected to have a comparable inhibitory spectrum with other reported lacticin 481-like lanthipeptides. The antimicrobial activity of estercin A was evaluated against Gram-positive strains using the agar diffusion method, showing a broad spectrum of activity (SI, Figure S3). Minimal inhibitory concentration (MIC) testing was then conducted on selected Gram-positive and pathogenic strains, with nisin as a control. The results indicated that estercin A demonstrated greater selectivity in its antimicrobial action compared to nisin (Table 2). Among these indicators, estercin A exhibits inhibitory activity against *S*. *aureus* strains, including a MRSA strain, with a MIC of 4 μg/mL, similar to that of nisin. In contrast, estercin A demonstrates low or no activity against tested *Enterococcus* and *Listeria* species. Usually, lanthipeptides are highly active against strains (excluding those harboring immunity-associated genes) from closely related species of the producers.^21^ As estercin A was identified from a *Clostridium* spp. strain, we suspected that perhaps it had great potential against important human pathogens from this genus. Among the three different *Clostridium* strains of our test list, estercin A showed much less inhibitory ability compared with nisin toward *C. botulinum* CECT551, with a MIC of 32 μg/mL, as opposed to 4 μg/mL for nisin. In contrast, it exhibited excellent activity against bowel infection-associated *C. perfringens* CECT376 and *C. tetani* CECT4629, achieving MICs of 1 μg/mL and 4 μg/mL, respectively, which is comparable with the activity of nisin.

**Table 2:** Minimal Inhibitory Concentration (MIC) of Estercin A and Nisin against Selected Gram-Positive Strains

	MIC (μg/mL)	
bacteria	estercin A	nisin
*Lactococcus lactis* MG1363	4	0.016
*Listeria monocytogenes* LMG10470	>64	8
*Staphylococcus aureus* LMG10147	16	16
*Staphylococcus aureus* LMG15975 (MRSA)	4	4
*Enterococcus faecalis* LMG16216 (VRE)	64	4
*Enterococcus faecium* LMG16003 (VRE)	64	4
*Bacillus cereus* ATCC14579	8	4
*Clostridium botulinum* CECT551	32	4
*Clostridium perfringens* CECT376	1	1
*Clostridium tetani* CECT4629	4	4

The comparison of the antimicrobial activity of estercin A and nisin reveals that estercin A is capable of potent and selective activity against clinically relevant pathogens such as *S. aureus* (MRSA), *C. perfringens*, and *C. tetani*. These are dangerous pathogenic bacteria frequently associated with multiple soft tissue infections.^37^ The antimicrobial spectrum also overlapped with that of some lacticin 481 family members but has unique features. For example, ruminococcin A was reported to have good activity against *C. perfringens* (MIC is 75 μg/mL) as well, which is much less active than estercin A (MIC is 1 μg/mL). This shows the superiority of applying antimicrobial peptides against pathogens from a closely related species of the producer. Our study also showed that compared with the best-studied lantibiotic nisin, estercin A has a narrower spectrum of antimicrobial activity, and thus has potential explicitly targeting pathogens and not effecting probiotic strains.

### Estercin A Exhibits Lipid II Binding Activity but Lacks Membrane Pore-Forming Ability

**Figure 5:**
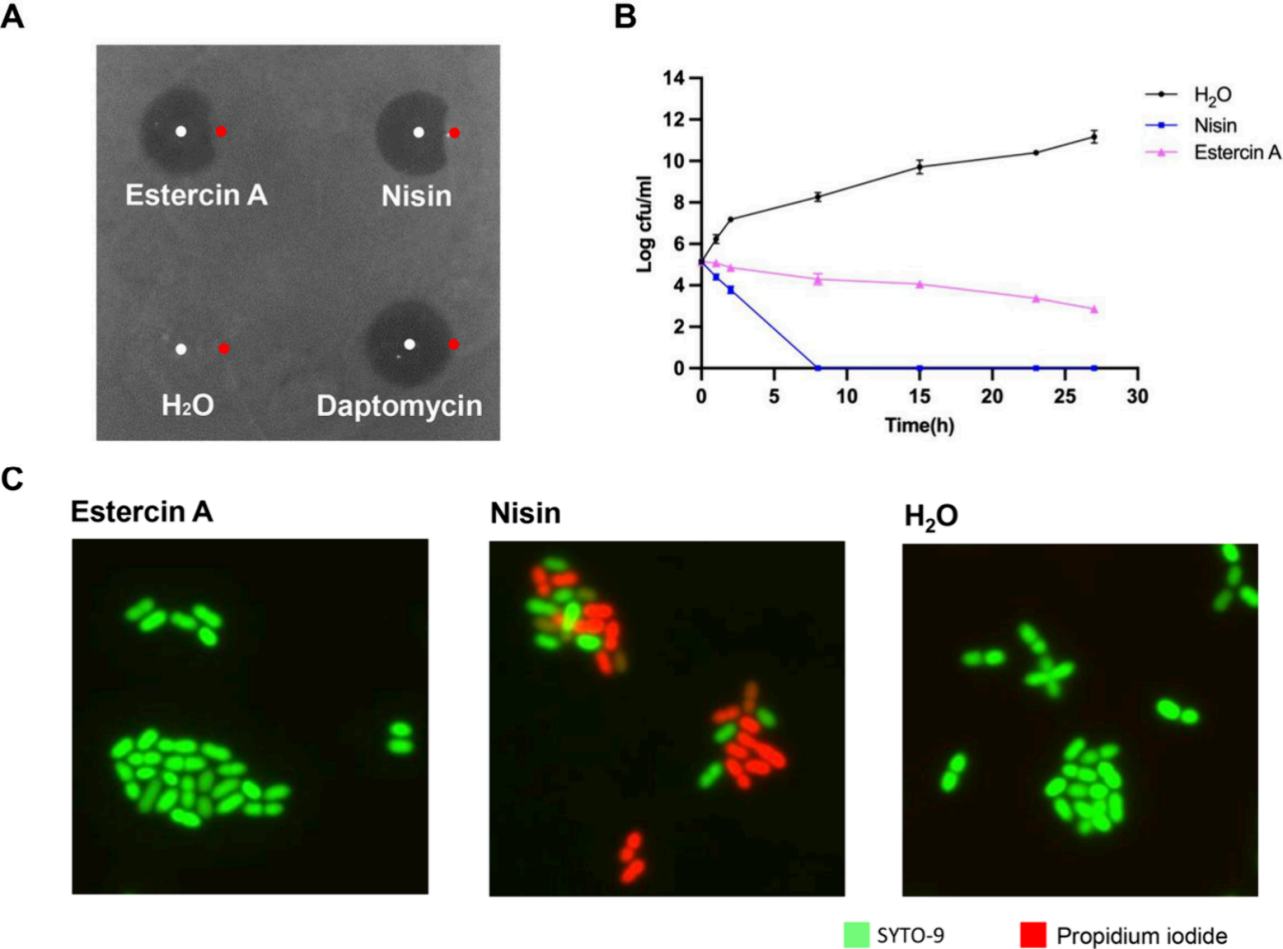
Antimicrobial mode of action of estercin A and nisin against *L*. *lactis* MG1363. Spot-on-lawn-based lipid II binding assay. Estercin A (8 μg) and nisin (2 μg) were spotted adjacent to lipid II (300 μM, 3 μL) and the antimicrobial activity determined. Daptomycin (2 μg) and H_2_O were used as controls (A). Time killing curves of estercin A and nisin against the bacterium. H_2_O was used as a control (B). Membrane pore forming ability of estercin A and nisin (2-fold MIC) in microbial cells were determined via fluorescence microscopy using the fluorescent dyes SYTO-9 (membrane permeable) and PI (membrane impermeable). H_2_O was used as a control (C).

An important mode of action of many class II lanthipeptides is the inhibition of bacterial cell wall synthesis by binding to lipid II, involving a special motif, e.g., the mersacidine-like lipid II binding motif.^38,39^ Estercin A also contains this motif including the characteristic amino acids 9Thr, 11Glu, and 12Cys (Figure 1B). To determine the mode of action of estercin A, its lipid II binding ability was investigated (Figure 5A). Indeed, estercin A binds to lipid II, as evidenced by a competition assay with lipid II against *L. lactis* MG1363. Nisin was used as a positive control, which is known to bind lipid II, and daptomycin whose antimicrobial activity does not involve lipid II binding was used as a negative control.

Another common mode of action for lanthipeptide is inhibiting target bacteria by membrane pore formation, and a typical example is nisin. We investigated whether estercin A also had pore-forming ability. We applied fluorescence microscopy using the green, fluorescent SYTO-9 and the red fluorescent propidium iodide (PI), to determine the pore-forming ability of estercin A (Figure 3C). SYTO-9 exhibits DNA binding and fluorescence emission by permeating both intact and compromised membranes, while PI exclusively binds to DNA and fluoresces upon penetration of damaged membranes.^40,41^ The red PI was observed after *L. lactis* MG1363 was exposed to nisin, which shows the cell membrane was incomplete, consistent with its membrane pore-forming ability. Conversely, only the green cells were observed when they were exposed to estercin A, demonstrating that the cell membrane was still intact. This suggests that estercin A cannot form pores on the membrane of target cells. Furthermore, pore formation typically correlates with the dissipation of the transmembrane electrostatic potential, resulting in membrane permeabilization and rapid cell death.^42^ The killing assay (Figure 5B) demonstrated rapid cell death when *L. lactis* MG1363 was treated with nisin. This observation was not made when estercin A was applied. The slower rate of killing observed for estercin A compared to nisin is consistent with its predicted mode of action: binding to lipid II without inducing membrane pore formation.

Lanthipeptides usually work through a variety of bactericidal mechanisms. Most reported lacticin 481-like lanthipeptides bind lipid II and further inhibit cell wall biosynthesis.^17,43,44^ Several members of the family, for example, streptococcin A-FF22 and nukacin ISK-1, can also form unstable pores in the cell membrane under certain conditions.^45,46^ We show that estercin A exerts lipid II binding ability, which may lead to the inhibition of cell wall biosynthesis of the target strains. However, our data indicates it lacks the ability to form membrane pores.

### Estercin A Displays High Thermal and pH Stability Comparable to Nisin

**Figure 6:**
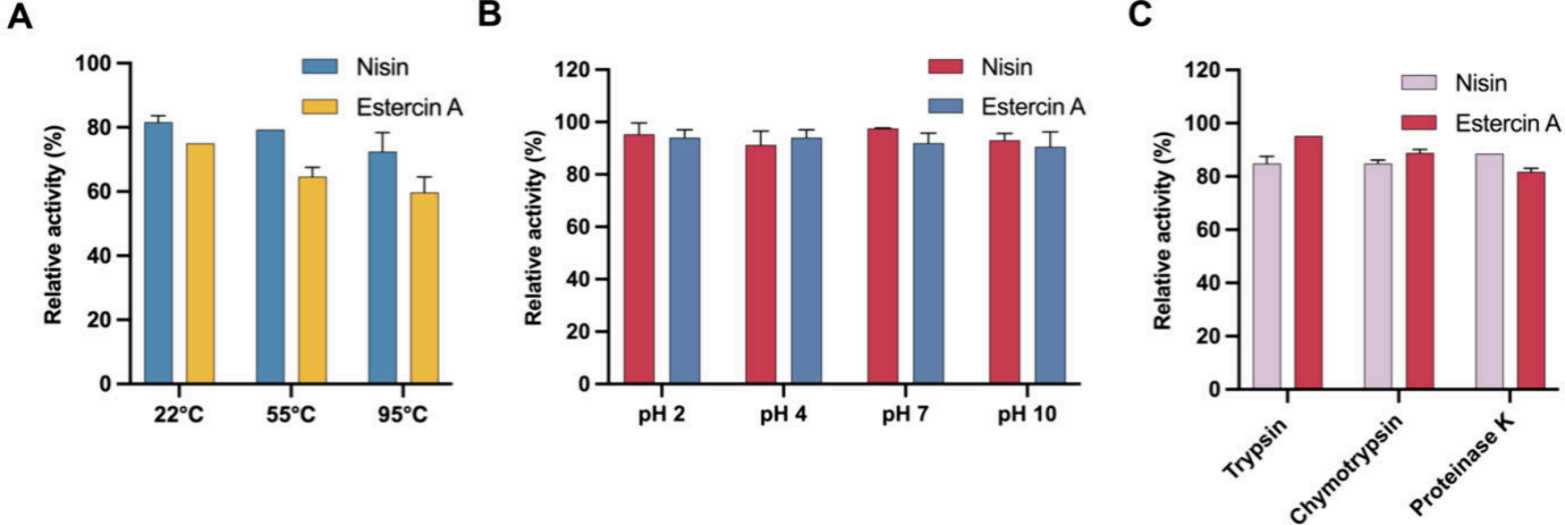
Thermal stability of estercin A compared to nisin (A). pH stability of estercin A compared to nisin (B). Relative remaining antimicrobial activity of the estercin A after exposure to different proteolytic enzymes (C).

The successful application of nisin as a food preservative is partly due to its stability.^12^ Because of this, we compared the stability of estercin A when exposed to different temperatures and pH values (Figure 6A,B). Both peptides were exposed for 4 h at 22 °C, 55 and 95 °C, and we found that estercin A was more sensitive than nisin when exposed to higher temperatures, but even with 95 °C exposure, the activity of estercin A was reduced by less than 30% (Figure 6A). Both nisin and estercin A are relatively stable at pH 2, 4, 7, or 10, as they retained most of their activity after the incubation (Figure 6B). Collectively, the data show that estercin A has a thermal and pH stability comparable to that of nisin.

A severe limitation of the application of lantibiotics is degradation by proteolytic enzymes. Here, the representative enzymes trypsin, chymotrypsin, and proteinase K were chosen for testing of enzymatic hydrolysis, and nisin was used as a comparison. The analysis showed estercin A and nisin had varying stability against these proteases (Figure 6C). Specifically, estercin A was more resistant to trypsin compared to nisin, which is consistent with the fact that estercin A lacks positively charged residues needed for trypsin digestion. As trypsin is a representative enzyme that exists in the small intestine to help us digest proteins, the outstanding stability of estercin A against trypsin makes it a prospective antibiotic for treating infections caused by enteropathogenic bacteria.

Trypsin, one of the most common proteases in nature, cleaves at the C-terminal side of Lys and Arg residues. Lys and Arg are the main positively charged residues and are confirmed to be necessary for membrane binding and biological activity, and are present in many lantibiotics (e.g., two in nisin, one in lacticin 481 and three in nukacin ISK-1).^17^ Protease sensitivity is one of the significant challenges for applying these lantibiotics. As estercin A contains no arginine and just one lysine as C-terminal residue, this explains the high resistance against trypsin degradation. The proteolysis result shows that compared with nisin, which contains two trypsin cleavage sites, estercin A is more resistant to trypsin degradation. This property makes it a promising candidate for replacing some trypsin sensitive lantibiotics, when administered via the gut.

### Unique Residues Associated with the Antibacterial Activity of Estercin A

**Figure 7:**
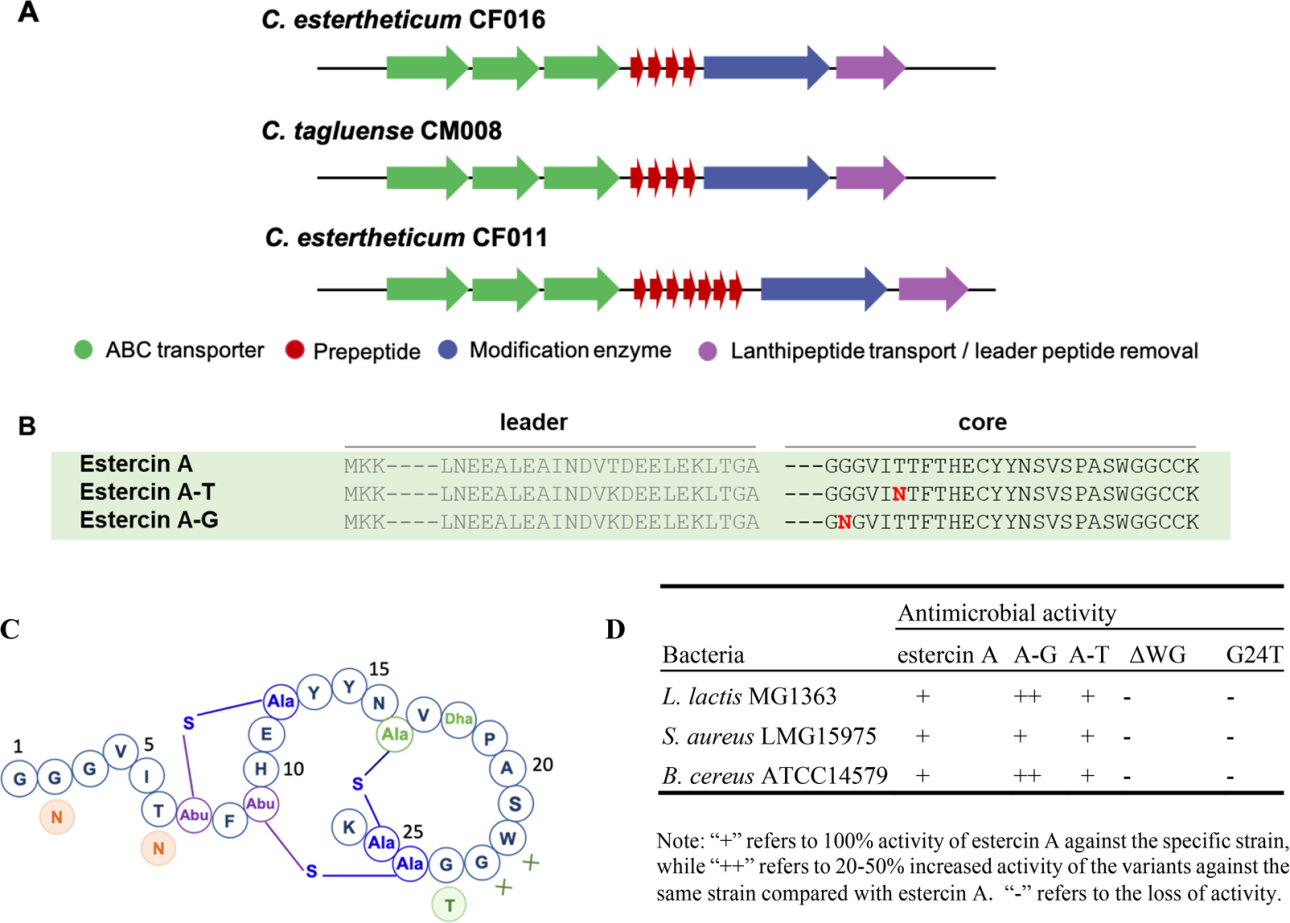
Biosynthetic gene cluster comparison of estercin A and two variants estercin A-T and estercin A-G identified within *Clostridium estertheticum* complex (CEC) (A). Sequence alignment of estercin A with natural variants (B). Natural and bioengineered analogues of estercin A with the substitution or deletion of amino acids (C). Activity of natural and bioengineering variants (D).

By exploring the genome mining result of the CEC strains, we found that estercin A has two natural variants.^16^ The lantibiotics estercin A-T and estercin A-G were identified from the genomes of *C. tagluense* CM008 and *C. estertheticum* CF011 separately (Figure 7A). They share the same leader part as estercin A and differ only in the sequence of the core peptides, i.e., the Thr6 is replaced by Asn in estercin A-T, and the Gly2 is replaced by Asn in estercin A-G (Figure 7B). To investigate the antimicrobial difference of these substitutes, we utilized the heterologous expression system to produce estercin A to obtain these natural variants. The mass result indicates that these variants displayed the same 4 dehydrations as in estercin A (SI, Figure S6B). Subsequently, the agar well diffusion test was done to compare the activity against selective strains (SI, Figure S6C). The results show that, in general, the natural variants feature a similar activity against the tested strains. However, estercin A-G was more active against *B. cereus* ATCC14579 than estercin A and estercin A-T.

The sequence alignment of estercin A and other lanthipeptides reveals it as a new member of the lacticin 481-like lantibiotic subgroup. They share similar ring patterns and the mersacidin-like lipid II binding motif (Figure 1B).^38^ However, the C-terminal part of estercin A is shorter and unique. Usually, the residue flanking an invariant double Cys is Thr instead of Gly, and the addition of Trp and Gly is also not common among the lacticin 481 family lanthipeptides. We investigated the importance of the unique parts by generating two bioengineered variants: ΔWG and G24T. The ΔWG mutant was generated through the deletion of Trp at position 22 and Gly at position 23. Similarly, the G24T mutant was produced by substituting Gly at position 24 with Thr. The variants were obtained using the heterologous expression system developed for the production of estercin A and analyzed. The MALDI-TOF MS analysis reveals that the mutant ΔWG installs three dehydrations, one dehydration less than in estercin A, suggesting that the modification machinery could not cope well with this deletion. For mutant G24T the same dehydration efficiency as for estercin A was observed (SI, Figure S7B). The antimicrobial activity test against several selective Gram-positive strains showed that both variants lost activity (SI, Figure S7C). This result indicates that the activity of estercin A is structurally related. Supporting evidence is that Gly has been reported to influence the structural conformation of lanthipeptides.^47,48^ Due to its small size and flexible characteristics, Gly residues often act as conformational hinges or linker segments within the lanthipeptide structure, allowing for flexibility and facilitating the adoption of specific conformations. These structural alterations can directly impact the biological activity of lanthipeptides, such as their antimicrobial activity.^47,48^ The bioengineered mutants demonstrate that the unique C-terminal part, being different from other lacticin 481 members, is crucial for the antimicrobial activity of estercin A.

In conclusion, the heterologous expression of estercin A and structure characterization are presented here and reveal that estercin A is a new member of the lacticin 481 family and harbors a unique and powerful C-terminal part. Estercin A exhibited selective activity against pathogenic strains, including *S*. *aureus*, *C*. *perfringens*, and *C. tetani.* Moreover, it is remarkably stable when exposed to challenging experimental conditions. These collective findings support estercin A as a promising candidate for therapeutic development.

## Experimental Section

### General Experimental Procedures

All primers were synthesized from Biolegio B.V. (Nijmegen, The Netherlands), while reagents for molecular biology experiments were obtained from Thermo Fisher Scientific (Waltham, MA, USA). Other chemical reagents were sourced from Sigma-Aldrich (St. Louis, MO, USA). *E. coli* Top10 and *E. coli* BL21(DE3) were used for plasmid maintenance and protein overproduction. Lipid II was synthesized and purified according to a previously published method and was kindly provided by Prof. Dr. E. J. (Eefjan) Breukink.^49^ All NMR data were collected at 298 K on a Bruker 600 MHz spectrometer. MALDI-TOF MS analyses were conducted on a 4800 MALDI TOF/TOF Analyzer in the linear-positive or reflector-positive mode, with α-cyano-4-hydroxycinnamic acid serving as the matrix. LC–MS was performed using a Thermo Scientific Q-Exactive mass spectrometer equipped with an Ultimate 3000 UPLC, an Acquity BEH C_18_ column (2.1 mm × 50 mm, 1.7 μm particle size, 200 Å; Waters).

### Bacterial Strains, Plasmids, and Growth Conditions

The strains and plasmids used in this study are listed in SI, Table S1. *E. coli* Top 10 was used as a host for plasmid construction. *E. coli* BL21(DE3) was used for the expression of peptides and proteins. The genome sequence was obtained from *C. estertheticum* CF016 for cloning the genes encoding the prepeptide (EstA), the modification enzyme (EstM), and the protease domain (EstTN150). All *E. coli* strains were cultured in Luria Broth (LB) broth at 37 °C, 220 rpm for growth and 18 °C, 220 rpm for the expression of peptides and proteins. 50 μg/mL kanamycin, 15 μg/mL chloramphenicol, or 100 μg/mL spectinomycin was added where necessary.

### Construction of pRSFduet-1 Derivatives for Coexpression of *estM* and *estA*

Primers used for cloning, mutations and sequencing in this work are listed in SI, Table S2. The gene encoding EstM harboring 15–20 bp overlapping ends with pRSFduet-1 multiple cloning sites was first amplified via PCR using genomic DNA of *C. estertheticum* CF016 as template and primers EstM-mcsII-Fw and EstM-mcsII-Rv. Linear pRSFduet-1 vector with 15–20 bp overlapping ends was amplified via PCR using circle pRSFduet-1 vector as a template and primers pRSFduet-mcsII-Fw and pRSFduet-mcsII-Rv. Subsequently the genomic PCR fragment was fused with the linear product of pRSFduet-1 vector using Gibson assembly to generate the pRSFduet-1/EstM vector. The DNA fragment encoding EstA harboring 15–20 bp overlapping ends with the pRSFduet-1 multiple cloning site 1 was then amplified using primers EstA-mcsI-Fw and EstA-mcsI-Rv. The linear pRSFduet-1/EstM vector was obtained through PCR amplification. The DNA fragment was then inserted into multiple cloning site 1 of the vector using Gibson assembly, generating the pRSFduet-1/His-EstA/EstM. Parallelly, pRSFduet-1/His-EstA and pCDFduet-1/His-EstTN150 were also constructed.

### Construction of Mutated Vectors for Expression and Purification of Variants of His6-EstA

We generated all the mutated plasmids via PCR using pRSFduet-1/His-EstA/EstM as a template and primers with designed mutations are listed in SI, Table S2. DpnI was added to the PCR reaction samples to digest the template DNA before the PCR products were transformed into the *E. coli* Top 10 cells. After overnight incubation, we picked several single colonies from selective plates to inoculate LB broth containing the appropriate antibiotics and extracted these mutated plasmids for DNA sequencing. The primers for DNA sequencing are listed in SI, Table S2. Finally, we obtained the mutated vectors: pRSFduet-1/His-EstA-T6N/EstM, pRSFduet-1/His-EstA-G2N/EstM, pRSFduet-1/His-EstAG24T/EstM, and pRSFduet-1/His-EstAΔWG/EstM.

### Peptide and Protein Expression

Expression of His-EstTN150, His-AprE, His-EstA, His-EstA/EstM, and the variants were conducted by the following protocol. For each, an overnight culture was grown from picked single colonies of freshly transformed *E. coli* BL21(DE3). Then, the overnight culture was diluted 50 times in 300 mL prewarmed LB broth and incubated for about 2.5 h at 37 °C, 220 rpm to reach a OD_600_ ≈ 0.5. The cultures were then put on ice for about 15 min to cool down, which was followed by adding 0.6 mM isopropyl β-d-1-thiogalactopyranoside (IPTG). Subsequently, the cultures were incubated for 18 h at 18 °C and 220 rpm for the peptide induction. After that, the cells were harvested at 8,000 rpm, with 20 min centrifugation and the supernatant was discarded.

### His-tag Purification

The peptides and proteins were first purified using His-tag purification. The harvested pellet was first washed with PBS buffer (137 mM NaCl, 2.7 mM KCl, 10 mM Na_2_HPO_4_, and 1.8 mM KH_2_PO_4_, pH 7.4), resuspended in binding buffer (20 mM NaH_2_PO_4_, 0.5 M NaCl, 20 mM imidazole, pH 7.4) and sonicated (VibraCell, 10 s ON, 10 s OFF, 80% amplitude, 20 min) on ice until the cells were shown visually to be lysed. The samples were centrifuged at 9,000 rpm for 1 h and the supernatant was collected. The supernatant was subsequently filtered using 0.45 μm filters. Next, the filtered supernatant was applied to an open column of 1 mL of Ni-NTA slurry prewashed by the binding buffer, washed with buffer (20 mM NaH_2_PO_4_, 0.5 M NaCl, 20 mM imidazole, pH 7.4), and eluted using 2 mL of elution buffer (20 mM NaH_2_PO_4_, 0.5 M NaCl, 250 mM imidazole, pH 7.4) per 1 mL of Ni-NTA slurry. The fractions were analyzed by Tricine-SDS-PAGE.

### C_18_ Purification and High-Performance Liquid Chromatograph (HPLC)

The collected eluted fractions were further desalted using open C_18_ columns. The columns were packed with 1 mL C_18_ resin (Waters) and prewashed with 100% acetonitrile (MeCN) (VWR) + 0.1% trifluoroacetic acid (TFA), and equilibrated with 100% Milli-Q + 0.1% TFA. The collected His-tag purified samples were transferred into the columns. After binding, the columns were first washed with 100% Milli-Q + 0.1% TFA followed by a second wash with 20% MeCN + 0.1% TFA. Finally, the samples were eluted with 4 mL of 40% MeCN + 0.1% TFA and freeze-dried.

For the further purification of the core peptides, the freeze-dried samples were dissolved in 2 mL Milli-Q water and incubated with 200 μL of the cleavage protease AprE for 3 h. The mixture was then filtered using a 0.2 μm filter before being injected into a 1260 Infinity HPLC system (Agilent) equipped with an Aeris 3.6 μm peptide XB-C_18_ column (Phenomenex). MeCN containing 0.1% TFA was served as the mobile phase, and a gradient of MeCN (25%–40%) over 30 min at 1 mL/min was applied for the peptide separation. The collected fractions were confirmed by MALDI-TOF MS and lyophilized for structural analysis (LC-MS/MS and NMR) and activity test. The core peptides were quantified using the NanoDrop (Thermo Scientific) calibrated with the extinction coefficient predicted by ExPASy (http://web.expasy.org/protparam/).

### Tricine-SDS-PAGE Analysis

The his-tagged purified precursor peptides were directly analyzed by Tricine-SDS-PAGE as previously described.^50^ 10 μL of each sample mixed with 10 μL 2× loading dye was heated at 95 °C for 10 min and then loaded on the gel (4% for stacking and 16% for separating). After running, the gel was stained with Coomassie Brilliant Blue G-250 buffer for about 1 h, followed by destaining in the destaining buffer (10% acetic acid, 50% methanol) for about 2 h.

### MALDI-TOF MS Characterization

Briefly, the freeze-dried samples from open C_18_ purification were dissolved in 1 mL Milli-Q, and 1 μL sample was spotted on the target plate, which was dried at room temperature. Subsequently, 1 μL of 5 mg/mL of a-cyano-4-hydroxycinnamic acid (50% MeCN + 0.1% TFA) was spotted on each sample, dried at room temperature, and then analyzed.^51^

### Detection of (Methyl-)Lanthionine Cyclization

The *N*-ethylmaleimide (NEM) assay to detect (non)cyclized Cys residues of estercin A was performed as follows. The samples were treated with 3 mM tris(2-carboxyethyl) phosphine (TCEP) in 50 mM 4-(2-hydroxyethyl)-1-piperazineethanesulfonic acid (HEPES) buffer (pH 7.0) for 30 min to ensure the complete reduction of free cysteines. Three mM freshly prepared NEM solution (50 mM in 100% ethanol) was then added to the mixture. The sample was left at room temperature for 30 min. Samples were further desalted by C_18_ zip-tip and checked by MALDI-TOF MS.^52^

### Liquid Chromatography–Tandem Mass Spectrometry (LC–MS/MS) Analysis

To get more information of the structure of estercin A, we performed a LC–MS/MS assay.^53^ For the UPLC part, the gradient was from 5% to 90% MeCN (0.1% formic acid (v/v)), running 60 min at a 0.3 mL/min flow rate. The following MS/MS part was performed in another separate run in PRM mode, selecting the different charged ions of the peptide for further analysis.

### NMR Analysis

NMR was used to study the details of the Lan/MeLan ring pattern. The chemical shift assignment was based on [1H,1H]-COSY, [1H,1H]-TOCSY, and [1H,1H]-NOESY spectra and supplemented by [13C,1H]-HSQC and [13C,1H]-HSQC-TOCSY experiments. All spectra were acquired at 298 K on a Bruker 600 MHz spectrometer equipped with a 5 mm triple resonance TCI z-gradient cryoprobe and analyzed using the software CcpNmr V3.^54^

### Agar Well Diffusion

Agar well diffusion growth-inhibition assays of peptides were conducted following a previously reported method.^55^ First, the peptides were dissolved in Milli-Q to achieve a concentration of 1 mg/mL using a published protocol.^41^ Agar plates were prepared by adding 20 μL of overnight cell culture to 20 mL of melted agar (LB or GM17 (M17 broth supplemented with 0.5% glucose)), cooled to about 42 °C at room temperature. The mixed agar was poured into a sterilized 100 mm round plate and solidified at room temperature for 20 min with 8 mm wells created. A sample of 8 μL of 1 mg/mL purified peptide was directly spotted on the well. The dried plates were incubated at 37 °C for 16 h, and the antimicrobial activity was determined by the presence or absence of zones of growth inhibition. The negative control was conducted using Milli-Q.

### Minimal Inhibitory Concentration (MIC) Test

MIC values of estercin A in comparison to nisin were evaluated by the doubling-dilution method according to the standard guidelines using cation-adjusted Mueller-Hinton broth (MHB).^55^ Briefly, the overnight bacterial culture was adjusted to approximately 5 × 10^5^ CFU/mL. MHB with bacteriocin was added to the first to the 10th wells to make the concentrations range from 0.02 μg/mL to 256 μg/mL. A positive control (MHB with bacterial inoculum but no bacteriocin) and a negative control (MHB without bacteria or bacteriocin) were included. The activity test of *Clostridium* strains was done using Reinforced *Clostridium* Medium (Sigma-Aldrich) and performed in a Coy Anaerobic Chamber following the same procedure mentioned. After 18 h of incubation at 37 °C (except for *L. lactis* MG1363 at 30 °C), the negative control was expected to remain clear (indicating no bacterial growth), while the positive control was turbid due to robust bacterial growth. The MIC was defined as the lowest concentration of bacteriocin with no visible growth in the well. All analyses were carried out with three biological replicates.

### Effects of pH, Enzyme, and Temperature on Antibacterial Activity

The effect of pH, proteolytic enzymes, and temperature on the antimicrobial activity of estercin A was carried out using the representative Gram-positive strain *L. lactis* MG1363 and compared with nisin. 8 μL of 1 mg/mL HPLC purified peptide mixed with proteolytic enzyme (final concentration of 1 mg/mL) was incubated at 37 °C for 2 h and was thereafter directly spotted on the agar plates (0.1%(v/v) overnight bacterial broth). The pH stability was determined by adjusting the pH of the samples to 2, 4, 7 and 10, using HCl or NaOH. The temperature stability was determined by incubating the peptides at 22 °C, 55 °C, 75 °C, and 95 °C for 4 h. All experiments were carried out with three biological replicates.

### Lipid II Binding Assay

lipid II was synthesized and purified as described in a previous study.^49^ The purified lipid II was stored in CHCl_3_/MeOH (2:1) at −20 °C until use.^51,56^ First, the GM17 agar plate with 0.1% (v/v) overnight culture of *L. lactis* MG1363 was prepared. The binding of the peptides to pure lipid II (0.6 M) was tested by the spot-on-the- lawn assay. Here, nisin and daptomycin were used for comparison.^51^

### Fluorescence Microscopy Assay

An overnight culture of *L. lactis* MG1363 was added to the GM17 broth to a final concentration of 1% (v/v) and grown to an OD_600_ of 0.8. The culture was centrifuged at 6,000 rpm for 5 min and washed three times in the MHB medium. Then, the cell density was normalized to an OD_600_ of 0.2 in MHB, and the tested peptides (a 2-fold MIC) were added. Simultaneously, 5 μM PI and SYTO 9 were added to the samples. After incubation at room temperature for 15 min, the samples were centrifuged, and the supernatant was removed. And the cells were washed three times with MHB. After that, the harvest cells were loaded on 1.5% agarose pads and analyzed by a DeltaVision Elite microscope (Applied Precision).

### Time Killing Assay of Estercin A Against *L. Lactis* MG1363

Time-killing assays were conducted according to the previously described protocol.^57^ Briefly, an overnight culture of *L. lactis* MG1363 was diluted 50-fold in GM17 and incubated at 30 °C. The cells were grown for about 3 h to an OD_600_ of 0.5, and then the cell concentration was adjusted to 5 × 10^5^ CFU/mL. Bacteria were then incubated with a concentration of 10-fold the MIC of estercin A or nisin. Untreated cells were used as a control. At predetermined time points, 50 μL was taken from each sample, and undiluted and 10-fold serially diluted suspensions were plated on GM17 agar plates. After the overnight incubation at 30 °C, the colonies on the plates were counted and then calculated as the colony-forming units per milliliter. The experiment was carried out in triplicate.

## Data Availability

The raw NMR data from this work has been deposited in the nmrXiv database (https://nmrxiv.org/) and is accessible at https://nmrxiv.org/P86.

## Supporting Information Available

MALDI-TOF spectra for results of His-EstA/EstM and after incubation with His-EstT150 or AprE *in vitro*; estercin A core peptide NOESY and TOCSY spectra used for structural assignment; zoomed overlay of TOCSY and NOESY spectra in the fingerprint region; MALDI-TOF spectra for results of prepeptides of His-EstA(S21A)/EstM and His-EstA(S24A)/EstM; antimicrobial activity screening assay of modified estercin A core peptide after cleavage of leader peptide using AprE; SDS-polyacrylamide gel electrophoresis, MALDI-TOF MS analysis and antimicrobial activity assay of the modified peptides (estercin A, estercin A-T, estercin A-G, ΔWG, and G24T); strains and plasmids used in this study (PDF)
