# Supplementary material for: Heterologous Expression and Characterization of Estercin A, a Class II Lanthipeptide Derived from Clostridium estertheticum CF016, with Antimicrobial Activity against Clinically Relevant Pathogens
Source: J Nat Prod. 2025 Jan 15;88(2):262–73. doi: 10.1021/acs.jnatprod.4c00814 (PMC11877530; doi:10.1021/acs.jnatprod.4c00814)
Supplement: Supplementary file 1 — np4c00814_si_001.pdf [file np4c00814_si_001.pdf]

Supporting Information

Heterologous expression and characterization of estercin A, a new Class II lanthipeptide derived from *Clostridium estertheticum* CF016, with potent antimicrobial activity against clinically relevant pathogens

Chenhui Wang,[†] Joseph Wambui,[‡] Maria Victoria Fernandez-Cantos,[†] Simon Jurt,[§] Jaap Broos,[†] Roger Stephan,[‡] and Oscar P. Kuipers[,†]*

†Department of Molecular Genetics, Groningen Biomolecular Sciences and Biotechnology Institute, University of Groningen, Groningen, The Netherlands

‡Institute for Food Safety and Hygiene, Vetsuisse Faculty, University of Zurich, Zurich, Switzerland

§Department of Chemistry, University of Zurich, Zurich, Switzerland

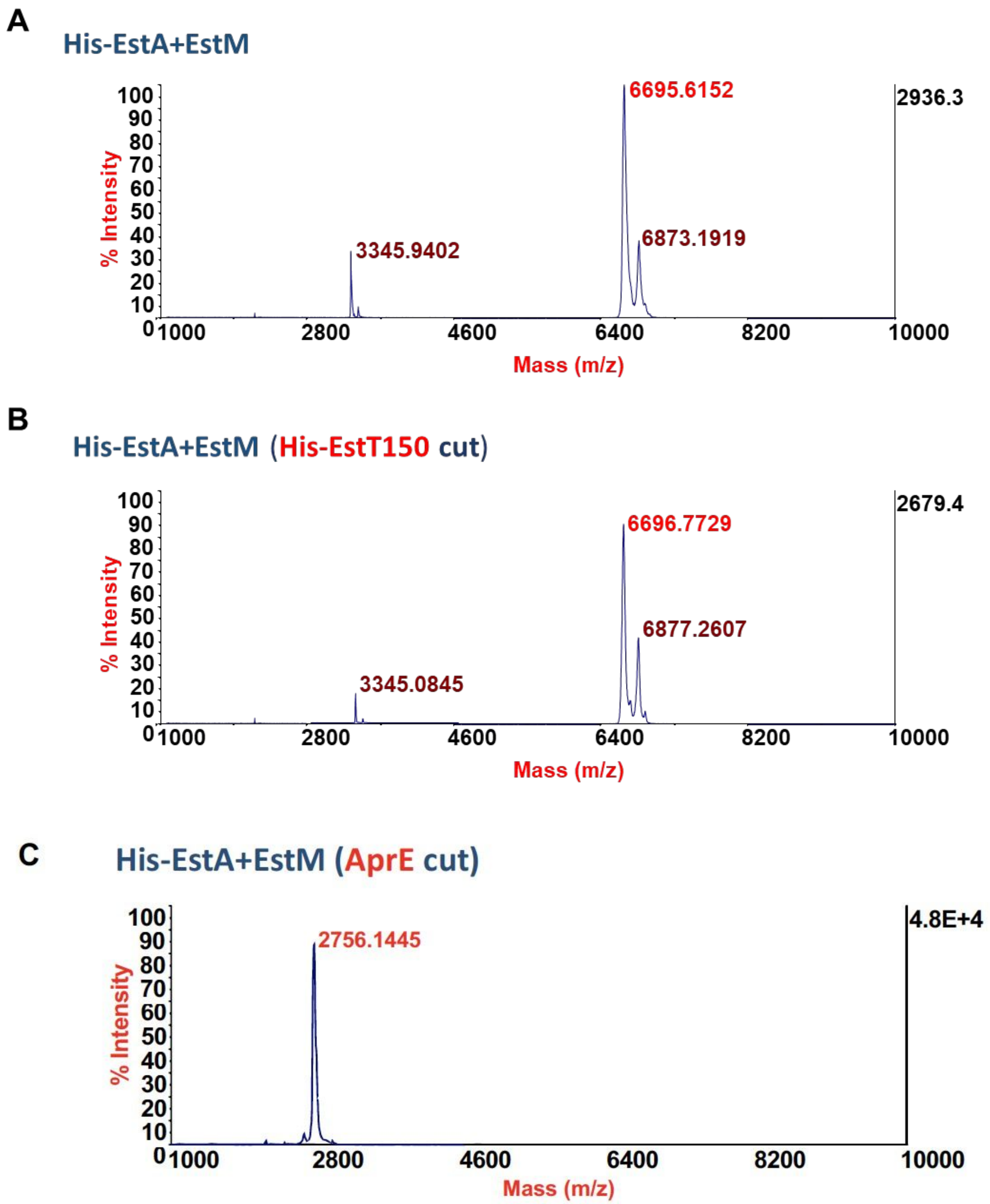

Figure S1. MALDI-TOF spectra for results of His-EstA/EstM (A) and after incubation with His-EstT150 (B) or AprE (C) *in vitro*.

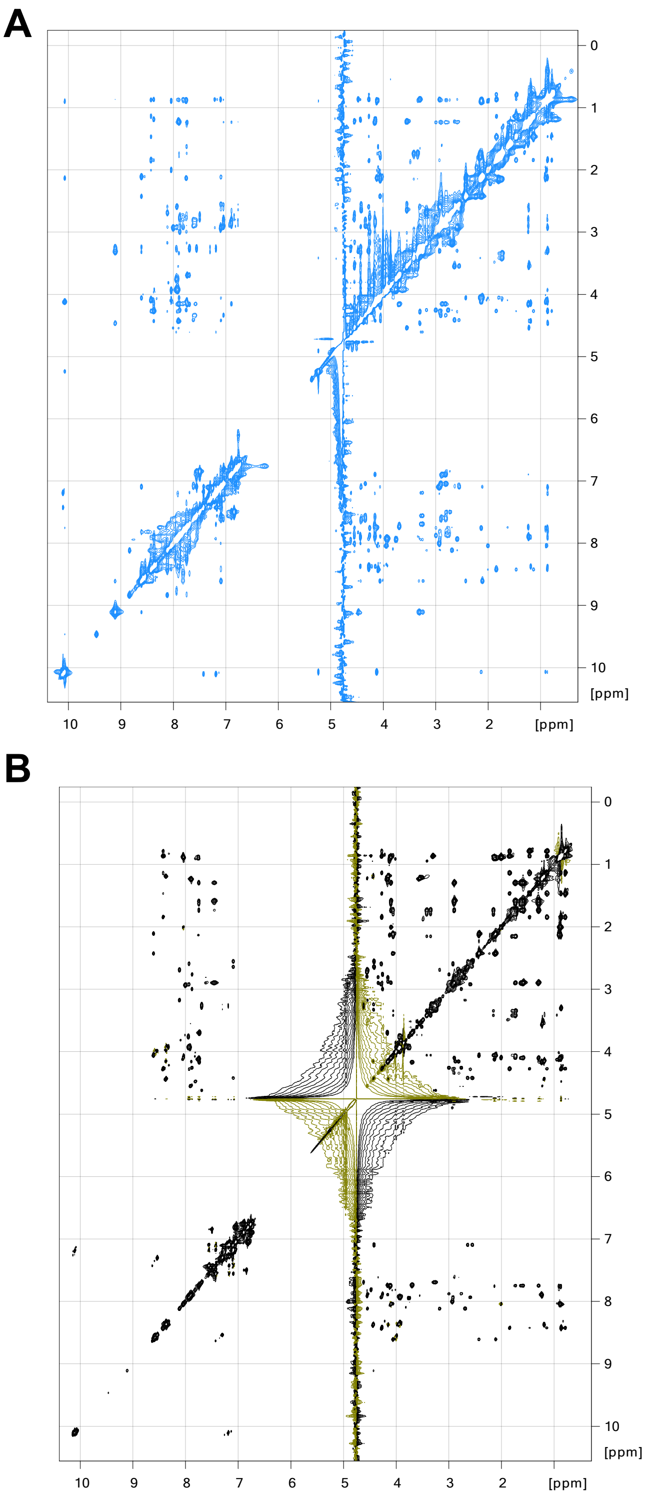

Figure S2. Estercin A core peptide NOESY(A) and TOCSY(B) spectra used for structural assignment.

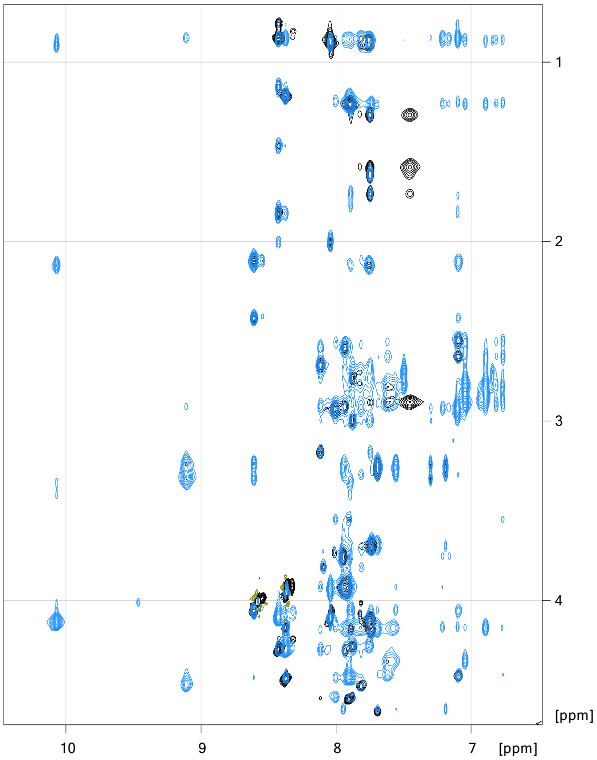

Figure S3. Zoomed overlay of TOCSY and NOESY spectra in the fingerprint region. The identified TOCSY spin systems were sequentially correlated by cross-referencing with the NOESY spectra.

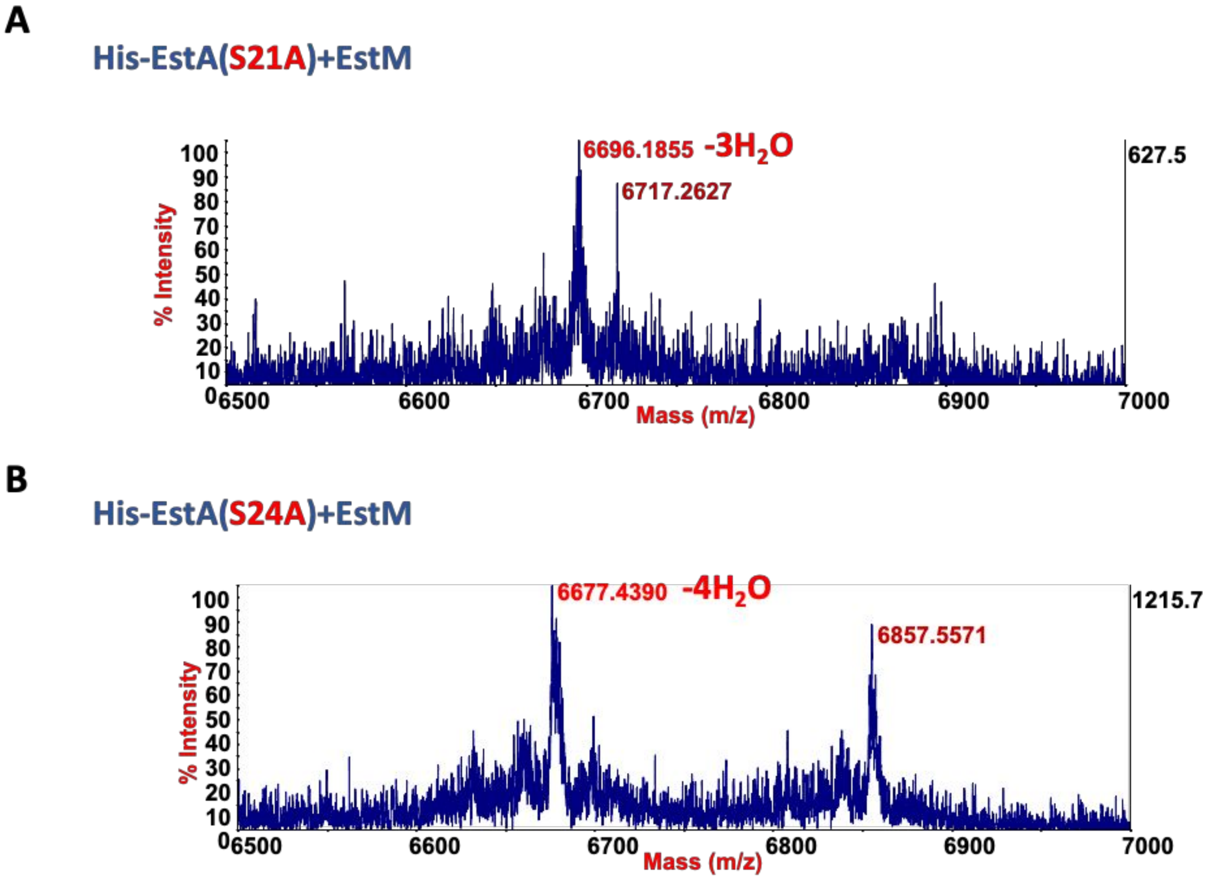

Figure S4. MALDI-TOF MS analysis of prepeptides of His-EstA(S21A)/EstM (A) and His-EstA(S24A)/EstM (B).

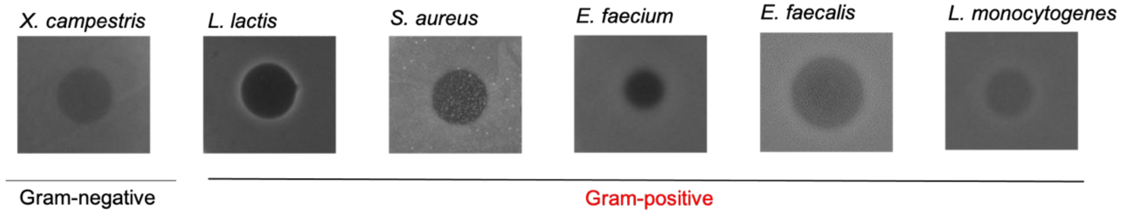

Figure S5. Antimicrobial activity screening assay of modified estercin A core peptide after cleavage of leader peptide using AprE

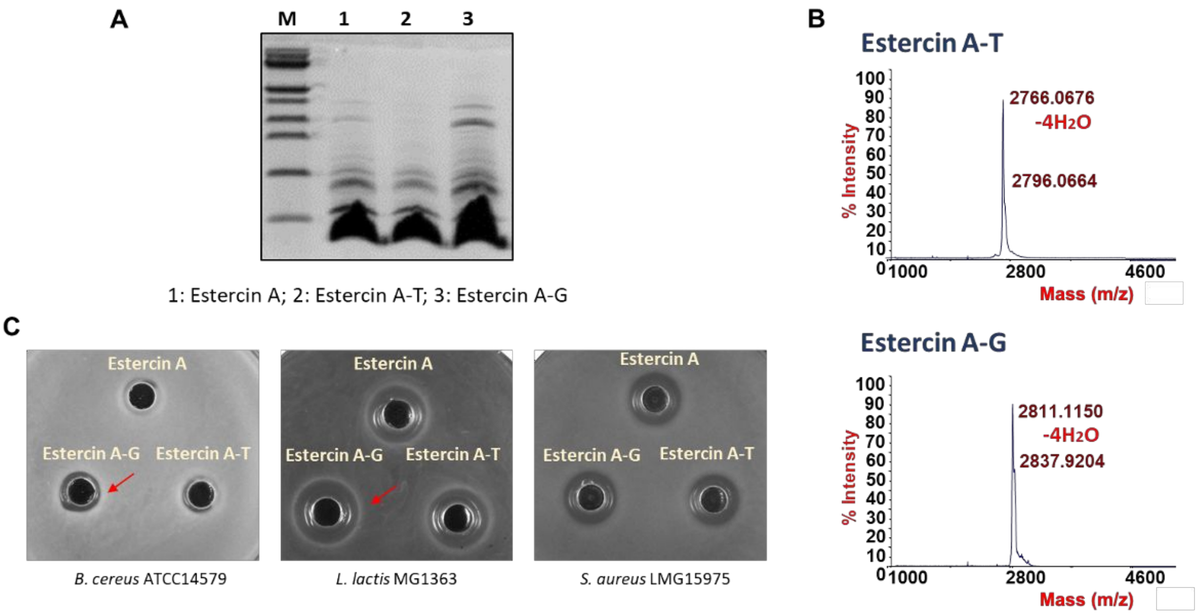

Figure S6. Tricine-SDS-PAGE of the modified prepeptides (estercin A, estercin A-T and estercin A-G) (A). MALDI-TOF MS analysis of these prepeptides (B). Antimicrobial activity assay of core peptides against selected Gram-positive strains (C).

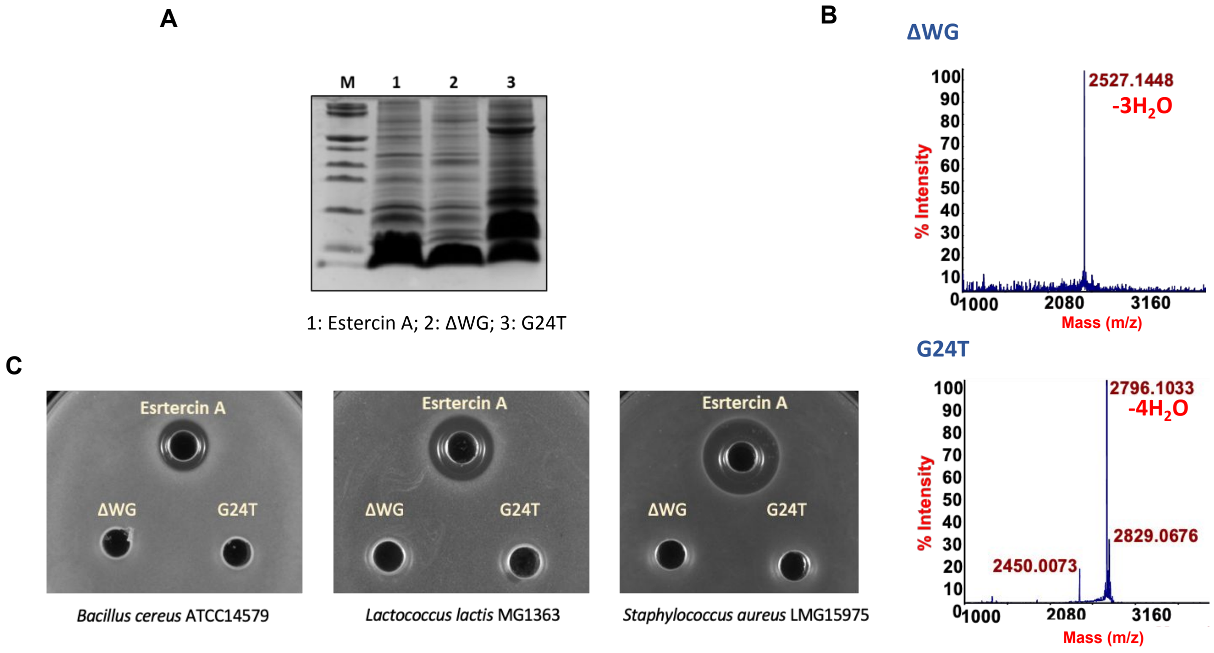

Figure S7. Tricine-SDS-PAGE of the modified prepeptides (estercin A, ΔWG and G24T) (A). MALDI-TOF MS analysis of these prepeptides (B). Antimicrobial activity assay of core peptides against selected Gram-positive strains (C).

Table S1. Strains and plasmids used in this study

**Strains or plasmids**	**Characteristics**	**Source**
**Strains**		
E. coli top10	Plasmid construction and plasmid maintenance	Invitrogen™
E. coli BL21(DE3)	Peptide expression	Thermo Scientific™
L. lactis MG1363	Indicator strain	MOLGEN Lab collection
Listeria monocytogenes LMG10470	Indicator strain	MOLGEN Lab collection
Bacillus cereus ATCC14579	Indicator strain	MOLGEN Lab collection
Staphylococcus aureus LMG10147	Indicator strain	MOLGEN Lab collection
S. aureus LMG15975	Indicator strain, MRSA	MOLGEN Lab collection
Enterococcus faecium LMG11423	Indicator strain	MOLGEN Lab collection
E. faecium LMG16003	Indicator strain, VRE	MOLGEN Lab collection
E. faecalis LMG16216	Indicator strain, VRE	MOLGEN Lab collection
Clostridium botulinum CECT551	Indicator strain	MOLGEN Lab collection
C. perfringens CECT376	Indicator strain	MOLGEN Lab collection

C. tetani<br>CECT4629	Indicator strain	MOLGEN Lab collection
C. estertheticum CF016	Template for replication of the precursor gene *estA* and the modification gene *estM*	[1]
**Plasmids**		
pRSFduet-1	Template for insertion of the precursor gene *estA* or/and modification gene *estM*	Novagen®
pCDFduet-1	Template for insertion of the gene *estTN150* involved in the leader peptide cleavage	Novagen®
pRSFduet-1/His-EstA	Encoding N-terminally His-tagged prepeptide of estercin A, under the control of T7 promoter	This study
pRSFduet-1/His-EstA/EstM	Encoding N-terminally His-tagged prepeptide of estercin A and modification enzyme EstM under the control of T7 promoter	This study
pRSFduet-1/His-EstA-T6N/EstM	estercin A mutation with the 6th threonine changed to asparagine on the core peptide	This study
pRSFduet-1/His-EstA-G2N/EstM	estercin A mutation with the 2nd glycine changed to asparagine on the core peptide	This study
pRSFduet-1/His-EstA-G24T/EstM	estercin A mutation with the 24th glycine changed to threonine on the core peptide	This study
pRSFduet-1/His-EstA-ΔWG/EstM	estercin A mutation with the deletion of tryptophan and glycine flanking the cysteine at the C-terminal end	This study
pRSFduet-1/His-EstA-S21A/EstM	estercin A mutation with the 21th serine changed to alanine on the core peptide	This study
pRSFduet-1/His-EstA-S24A/EstM	estercin A mutation with the 24th serine changed to alanine on the core peptide	This study

pCDFduet-1/His-EstTN150	Encoding N-terminally His-tagged proteolytic domain of EstT, EstTN150	This study
pDR111/His-AprE	Encoding His-tagged *B. amyloliquefaciens* BH072 protease AprE	[2]

Table S2. Primers for PCRs used in this study

**Name**	**Templates**	**primers**	**Nucleic acid sequences (5' to 3')**	**Characteristics (5'-lable)**
pRSFduet-1/His-EstA	*C. esterthеticum* C F016 genomic DNA	EstA-mcsI-Fw	GCAGCAGCCATCACCATCATCACCACATGA AAAAATTAAA TGAAGAAGC	
		EstA-mcsI-Rv	GAATTCGGATCCTGGCTTTATTTGCAACAGC CACCCCAGC	
	pRSFduet-1	pRSFduet-mcsI-Fw	CAGCATAAGCAGATCTCAATTGGATATCGGC	
		pRSFduet-mcsI-Rv	ATGGTGATGGCTGCTGCCCATG	
pRSFduet-1/His-EstA/EstM	*C. esterthеticum* C F016 genomic DNA	EstM-mcsII-Fw	GATATACATATGAGTATTAAAGATTTAATCA AGG	

		EstM-mcsII-Rv	CAATTGAGATCTGCTTATGCTGCTGGTCCTTCTAAAA	
	pRSFduet-1/His-EstA	pRSFduet-mcsII -Fw		
		pRSFduet-mcsII -Rv		
pCDFduet-1/His-EstTN150	*C. esterthetucum* CF016 genomic DNA	EstTN150-mcsI-Fw	CATGGGCAGCAGCCATCACCATCATCACCACATGATATTG CAAAATATGGCATTAAAA	
		EstTN150-mcsI-Rv	CTCGAATTCGGATCCTGGCTTTACTCAGTATGCTGCTCAA AATCATC	
	pCDFduet-1	pCDFduet-mcsI-Fw	AGCCAGGATCCGAATTCGAGCTC	
		pCDFduet-mcsI-Rv	GGTGATGGCTGCTGCCCATGG	
pRSFduet-1/His-EstA-T6N/EstM	pRSFduet-1/His-EstA/EstM	T6N-Fw	ATACATTTACACACGAATGCTACTATAATTC	5'-phosphorylation

		T6N-Rv	TTATAACTCCACCGCCTGCTCC	
pRSFduet-1/His-EstA-G2N/EstM	pRSFduet-1/His-EstA/EstM	G2N-Fw	AATGGAGTTATAACTACATTTACACACGAATGC	5'-phosphorylation
		G2N-Rv	GCCTGCTCCTGTTAATTTTTCTAATTCC	
pRSFduet-1/His-EstA-G24T/EstM	pRSFduet-1/His-EstA/EstM	G24T-Fw	ACATGTTGCAAATAAAGCCAGGATCCG	5'-phosphorylation
		G24T-Rv	ACCCCAGCTAGCTGGTGATAC	
pRSFduet-1/His-EstA-ΔWG/EstM	pRSFduet-1/His-EstA/EstM	ΔWG-Fw	GGCTGTTGCAAATAAAGCCAGG	5'-phosphorylation
		ΔWG-Rv	ACCCCAGCTAGCTGGTGATAC	
pRSFduet-1/His-EstA-S21A/EstM	pRSFduet-1/His-EstA/EstM	S21A-Fw	GCACCAGCTAGCTGGGGTGGCTGTTGC	5'-phosphorylation
		S21A-Rv	TACTGAATTATAGTAGCATTCGTGTG	
pRSFduet-1/His-EstA-S24A/EstM	pRSFduet-1/His-EstA/EstM	S24A-Fw	GCATGGGGTGGCTGTTGCAAATAAAGCC	5'-phosphorylation
		S24A-Rv	AGCTGGTGATACTGAATTATAGTAGC	

(1) Wambui, J.; Stevens, M. J. A.; Sieber, S.; Cernela, N.; Perreten, V.; Stephan, R. Targeted Genome Mining Reveals the Psychrophilic Clostridium estertheticum Complex as a Potential Source for Novel Bacteriocins, Including Cesin A and Estercticin A. *Front Microbiol* **2021**, *12*, 801467.
(2) Viel, J. H.; Jaarsma, A. H.; Kuipers, O. P. Heterologous Expression of Mersacidin in Escherichia coli Elucidates the Mode of Leader Processing. *ACS Synth Biol* **2021**, *10* (3), 600-608.
